# Editorials

**Published:** 1910-12

**Authors:** 


### EDITORIALS

**The Business Office of the *JOURNAL-RECORD* is 1 1-2 to 5 1-2 South Broad Street.**

The Editorial Office is 1014-15 Century Building.

Address all Business Communications to *Journal-Record of Medicine*, 1 1-2 to 5 1-2 South Broad Street.

Make remittances payable to *THE JOURNAL-RECORD OF MEDICINE*.

On matters pertaining to the Editorial and Original Communications, address Edgar G. Ballenger, M. D., Atlanta, Ga.

Reprints of Original Articles will be furnished at cost price. Requests for the same should always be made in THE MANUSCRIPT.

We will present, postpaid, on request, to each contributor of an original article, **twenty (20) marked copies of *THE JOURNAL-RECORD OF MEDICINE* containing such article.**

### DEATH OF DR. ABNER W. CALHOUN.

Probably no living men in the South was more widely and more favorably known than was Dr. Abner W. Calhoun, whose death occurred September the 22nd, after a lingering illness. Dr. Calhoun was one of the first eye, ear and nose specialists in this part of the country and by careful, conscientious work developed an etensxive and lucrative practice in his chosen specialty. It is doubtful if there is a village so small in the Southern States to which Dr. Calhoun's reputation has not extended. He was born in Newnan, Georgia, April 16th, 1845, and when nearly sixteen years of age enlisted in the Southern army where he served until the close of the Civil War.

After being graduated from the Jefferson Medical College and two years' study abroad, Dr. Calhoun associated himself with Dr. Willis Westmoreland, Sr. Later this association was severed, and Dr. Calhoun began his successful career as a specialist. His first office was at the corner of Broad and Alabama steeets, later he moved to Marietta Street, and finally to the Candler building. Dr. Calhoun was a member of the various county, State and National Medical Associations, which he attended until his latter years. He also contributed not infrequently to Medical Journals.

In the death of Dr. Calhoun Atlanta has lost one of her best and most influential citizens and the South one of her "master surgeons."

Dr. Calhoun's practice has been continued by his son Phinizy, who has ably assisted in this work for a number of years. It may truly be said of him that he is a son of whom his father may be justly proud.

---

### CONSERVATION OF BABIES.

The interest recently manifested in the protection and care of infants and children will probably be productive of much more good than has been so much of the agitation against the so-called race suicide. The recent meeting in Baltimore aroused much enthusiasm and we anticipate much good work from this organization. The joint meeting of the Fulton County Medical Society and the Women's Clubs, Dec. 1st, to discuss this subject was very well attended and two excellent papers were read by Drs. Chas. Boynton and Stewart Roberts.

Dr. Boynton spoke chiefly upon the things which concern the health of the infant, while Dr. Roberts considered the child —a human being which grows—and showed the excessive demand placed upon them. The free discussion which followed was clearly in favor of changes being made in our school system. The children and teachers both, as a rule, are overworked trying to crowd into a little mind much useless information, while many essential things are never taught. Some day we will see many changes in our school system.

---

### TAKE NOTICE.

On January 10th and 11th next, the Chattahoochee Valley Medical and Surgical Association will meet in the goodly city of Columbus, Georgia.

The indefatigable secretary, Dr. Love, has prepared a program which would do credit to a State meeting, for in that program are found the names of some of our brightest representatives in Georgia and Alabama—men whose presence would grace any occasion, whose words of wisdom would instruct any scientific body.

As to the material welcome held in store by the fraternity of Columbus, we are informed that it will be something unique in the annals of medical entertainment, and that it will not be excelled even by the ancient celebrations held at Epidaurus on the coasts of Laconica, where Aesculapius and his fair daughter Hygieia were welcomed with the sound of the cornet, the harp, the sackbut, the psaltery and the dulcimer.

Under these circumstances, therefore, we commnd to the brethren that, laying aside all business, professional or otherwise, they betake themselves to Columbus at the appointed time.

They will surely not regret it.

